# Immunogold FIB-SEM: Combining Volumetric Ultrastructure Visualization with 3D Biomolecular Analysis to Dissect Cell–Environment Interactions

**DOI:** 10.1002/adma.201900488

**Published:** 2019-06-13

**Authors:** Sahana Gopal, Ciro Chiappini, James P. K. Armstrong, Qu Chen, Andrea Serio, Chia-Chen Hsu, Christoph Meinert, Travis J. Klein, Dietmar W. Hutmacher, Stephen Rothery, Molly M. Stevens

**Affiliations:** Department of Materials, Department of Bioengineering and Institute of Biomedical Engineering Imperial College London, London, SW7 2AZ, UK; Department of Medicine Imperial College London London, W12 0NN, UK; Department of Materials, Department of Bioengineering and Institute of Biomedical Engineering Imperial College London, London, SW7 2AZ, UK; Centre for Craniofacial and Regenerative Biology King’s College London, London, SE1 9RT, UK; Department of Materials, Department of Bioengineering and Institute of Biomedical Engineering Imperial College London, London, SW7 2AZ, UK; Department of Materials, Department of Bioengineering and Institute of Biomedical Engineering Imperial College London, London, SW7 2AZ, UK; Centre for Craniofacial and Regenerative Biology King’s College London, London, SE1 9RT, UK; Department of Materials, Department of Bioengineering and Institute of Biomedical Engineering Imperial College London, London, SW7 2AZ, UK; Institute of Health and Biomedical Innovation Queensland University of Technology Brisbane, Queensland 4059, Australia; Institute of Health and Biomedical Innovation Queensland University of Technology Brisbane, Queensland 4059, Australia; Australian Research Council Industrial Transformation Training Centre Queensland University of Technology, Brisbane, Queensland 4059, Australia; Facility for Light Microscopy Imperial College London London, SW7 2AZ, UK; Department of Materials, Department of Bioengineering and Institute of Biomedical Engineering Imperial College London, London, SW7 2AZ, UK

**Keywords:** 3D reconstruction, electron microscopy, FIB-SEM, image analysis, immunogold

## Abstract

Volumetric imaging techniques capable of correlating structural and functional information with nanoscale resolution are necessary to broaden the insight into cellular processes within complex biological systems. The recent emergence of focused ion beam scanning electron microscopy (FIB-SEM) has provided unparalleled insight through the volumetric investigation of ultrastructure; however, it does not provide biomolecular information at equivalent resolution. Here, immunogold FIB-SEM, which combines antigen labeling with in situ FIB-SEM imaging, is developed in order to spatially map ultrastructural and biomolecular information simultaneously. This method is applied to investigate two different cell–material systems: the localization of histone epigenetic modifications in neural stem cells cultured on microstructured substrates and the distribution of nuclear pore complexes in myoblasts differentiated on a soft hydrogel surface. Immunogold FIB-SEM offers the potential for broad applicability to correlate structure and function with nanoscale resolution when addressing questions across cell biology, biomaterials, and regenerative medicine.

It is increasingly evident that interactions between cells and their extracellular environment are not only dependent on biochemical signals, but also rely on biophysical interactions. Indeed, cells alter their phenotype in response to electrical stimuli,^[1]^ material topography,^[2]^ substrate stiffness,^[3]^ or externally applied forces, such as shear,^[4]^ compression,^[5]^ and tension.^[6]^ In order to design and implement successful strategies for guiding cell behavior, we must be able to dissect the underlying mechanisms that regulate cellular responses. However, since cellular processes are mediated by molecular interactions occurring primarily at the nanoscale, their investigation requires analytical tools capable of mapping biomolecular information with high spatial resolution. The development of advanced optical imaging techniques, such as structured illumination microscopy (SIM), photoactivated localization microscopy (PALM), stimulated emission depleted microscopy (STED), and stochastic optical reconstruction microscopy (STORM), has enabled super-resolution microscopy of biological systems. These imaging modes, however, are challenged in observing overall cell ultrastructure due to the limited multiplexing of targets and the increased optical aberrations at greater sample imaging depth. For instance, PALM and STORM offer the highest lateral resolution of 10 nm but are limited to the visualization of fluorophores less than 300 nm from the sample surface.^[7]^ Since STED microscopy is based on a confocal set-up, it offers greater depth of imaging but is accompanied by low out-of-plane resolution and high laser power requirements that accelerate photobleaching.^[8]^ The need for 3D reconstruction for SIM, the stochastic nature of STORM, and contrast enhancements required for STED complicate the data acquisition process for generating volumetric high-resolution ultrastructural information.^[9,10]^

The recent emergence of focused ion beam scanning electron microscopy (FIB-SEM), in which samples are milled and sequentially imaged using electron microscopy, offers the potential for unsurpassed in-plane (≈1 nm) and out-of-plane (10 nm) resolution.^[11]^ Since FIB-SEM is a slice-and-view technique, it has a practical resolution that is limited by the time, field of view, and milling current required to image the volume of interest. Nevertheless, FIB-SEM can be used to reconstruct and visualize large volumes of cell ultrastructure in three dimensions (3D) and can be used to investigate nanoscale processes at the cell–material interface by in situ milling at particular regions of interest. The ability to rapidly overview the cell–material interface over large areas by SEM imaging enables informed and accurate cell selection prior to milling. Such approaches have provided unprecedented insight into the ultrastructural changes that can occur as cells interface with a material;^[12–15]^ however, conventional FIB-SEM still does not provide any biomolecular information (e.g., protein localization). This limitation can be partially addressed by correlative techniques,^[16]^ in which samples are sequentially imaged by optical microscopy and FIB-SEM, and then superimposed to generate a reconstructed map. This technique, however, is labor intensive, extremely low throughput, and can have low yield due to sample loss. Most importantly, the quality of the biomolecular mapping is limited by the resolution of the optical microscopy, which remains at least one order of magnitude lower than electron microscopy, even for super-resolution techniques.

Here, we report a strategy for nanoscale volumetric biomolecular mapping using FIB-SEM imaging of immunogold-labeled cells. Immunogold labeling, which involves labeling antigens with gold-conjugated antibodies, is a well-established strategy used to correlate biomolecular and structural information with high spatial resolution. It is commonly used in conventional transmission electron microscopy (TEM) analysis, for instance, to dissect the precise subcellular localization of pollutant nanoparticles across pulmonary cells and tissues^[17]^ or to localize nuclear pore complex (NPC) proteins that regulate nucleocytoplasmic movement.^[18]^ However, it is important to note that fixation, permeabilization, and immunolabeling procedures can lead to certain imaging artifacts. For instance, the use of methanol or acetone for fixation and permeabilization without the prior application of formaldehyde can drastically alter cell ultrastructure.^[19]^ In addition, membrane proteins may also be extracted by permeabilization agents such as Triton X-100 after fixation with formaldehyde, highlighting the need to optimize specific protocols for certain proteins of interest.^[20]^ Moreover, fluorescently labeled gold nanoparticles that are routinely used in immunolabeling for correlative light electron microscopy (CLEM) have been shown to dissociate under certain conditions, leading to poor co-localization between the probe and the target.^[21]^

Specifically, investigating how ultrastructural variations in nuclear morphology correlate with changes in the arrangement of nuclear biological regulators of gene expression remains elusive due to the resolution limits of current analytical techniques. A better insight into the modulation of their localization could improve our understanding of the regulatory processes underlying cell–environment interactions. We addressed this key challenge by combining the volumetric nanometer scale structural information of FIB-SEM imaging with the biomolecular information provided by immunogold labeling. In particular, we used immunogold FIB-SEM to study the role of topography and differentiation on the nanoscale spatial distribution of epigenetic marks and nuclear pore complex proteins in two independent cell–material systems. We show that during the differentiation of neural stem cells on microgrooved surfaces, specific epigenetic marks associated with gene silencing favor the nuclear periphery, while myogenesis of myoblasts on a hydrogel substrate is accompanied by localization of NPCs to sites of nanoscale invaginations in the nucleus. These examples illustrate the utility of immunogold FIB-SEM in investigating how ultrastructural variations in nuclear morphology correlate with changes in the spatial arrangement of biological regulators of gene expression; key mechanistic questions that current analytical techniques have been thus far unable to answer.

In order to incorporate immunogold labeling with FIB-SEM, we developed a workflow combining sample preparation processes from immunofluorescence and electron microscopy (Figure 1a–f). We fixed and permeabilized cells and then immunolabeled with primary antibodies for the antigen of interest (Figure 1a), and then stained with Fab’ fragment secondary antibodies conjugated to both a fluorophore and a 1.4 nm diameter gold nanoparticle (Figure 1b). The conjugated fluorophore enabled rapid quality control by fluorescence microscopy, allowing us to prescreen and optimize conditions prior to FIB-SEM. Following fluorescence imaging, the gold nanoparticles were catalytically enhanced to a diameter of 20–30 nm (Figure 1c), before the samples were stained, resin-embedded, and thin-layer plasticized for FIB-SEM imaging (Figure 1d). The serial cross-sectional images obtained using FIB-SEM were then aligned, and regions of interest (such as the nucleus and immunogold labels) were segmented (Figure 1e) and analyzed in terms of volume, co-localization, and marker separation distance (Figure 1f).

We first optimized sample preparation in order to yield specific antigen recognition while preserving the native ultrastructure of the processed cells. To this end, we immunolabeled the nuclear epigenetic mark H3 lysine 9 trimethylation (H3K9me3) in neural stem cells that had been permeabilized with either Triton X-100 or Saponin. Under both conditions, widefield fluorescence microscopy showed H3K9me3 marks co-localized with 4′,6-diamidino-2-phenylindole (DAPI) in the cell nucleus, confirming that each agent had successfully permeabilized the nuclear membrane (Figure 2a). Using FIB-SEM, we observed bright, punctate 20–30 nm spots within the nuclei of immunolabeled cells. We used elemental analysis by energy-dispersive X-ray (EDX) spectroscopy to confirm that these spots contained gold, suggesting successful immunolabeling and catalytic nanoparticle enhancement (Figure 2b). We did not observe any gold nanoparticles in the cells prepared without any primary antibody, suggesting that the immunogold labeling process was highly specific to the presence of the primary antibody in the sample (Figure 2b; Section S1, Supporting Information).

FIB-SEM imaging, however, revealed a broad difference in ultrastructure preservation for the two permeabilization strategies. Most notably, the cells permeabilized with Triton X-100 exhibited widespread ultrastructural damage in the form of fragmented vesicles, empty space, and lack of visible organelles in the cytosol, none of which were present in the nonpermeabilized controls (Figure 2c). Conversely, the Saponin-permeabilized cells exhibited intact vesicles, vacuoles, and other intracellular structures, with the overall nuclear architecture bearing great similarity to the nonpermeabilized cells. Moreover, we also observed a high proportion of cytosolic immunolabeling in cells permeabilized by Triton X-100 (38% ± 6%) despite using a primary antibody for H3K9me3, a nuclear antigen. This nonspecific cytosolic immunolabeling could be significantly reduced by using Saponin permeabilization (4% ± 2%) (Figure 2d). Taken together, these results indicate that our sample preparation route along with the use of Saponin permeabilization can provide specific immunogold labeling and well-preserved cell ultrastructure.

Having established a specific immunogold FIB-SEM workflow for nuclear antigens, we sought to precisely map the volumetric distribution of the epigenetic mark H3K9me3 within the nucleus of cells subjected to different biophysical cues. Specifically, we compared immunolabeling in neural stem cells cultured for 2 days on either flat polydimethylsiloxane (PDMS) substrates or PDMS surfaces textured with 10 μm wide and 10 μm deep microgrooves (Figure 3a). It is known that confining cells within microgrooves can modulate epigenetic changes such as methylation and acetylation,^[22,23]^ but there is limited information regarding the spatial arrangement of this remodeling process, largely due to the limited availability of analytical methods. For instance, we know that chromatin condensation and relocation to the nuclear periphery is often associated with gene silencing;^[24]^ however, the reported histone epigenetic modifications that putatively associate with such gene active/inactive regions have never been visualized directly. Using immunogold FIB-SEM imaging and 90 nm thick serial cross sections, we were able to map H3K9me3 distribution in 3D across whole nuclei (Figure 3b). We obtained a 90 nm slice thickness using a nominal milling thickness of 30 nm and imaging every third section. The FIB-SEM volumetric reconstruction revealed striking nuclear shape differences between the two groups, which we quantified using sphericity measurements (where a perfect sphere has a value of *S* = 1). This image analysis revealed that the nuclei of cells cultured on microgrooves were significantly more elongated (*S* = 0.25 ± 0.04) than the nuclei of cells grown on flat substrates (*S* = 0.40 ± 0.07), while the volume of these nuclei remained unaltered (Figure 3c,d). We also counted the number of H3K9me3 immunolabels in each group, measuring a 1.4-fold increase for the nuclei of cells cultured on microgrooves compared to those grown on flat substrates (Figure 3e). The volumetric nanoscale functional information provided by immunogold FIB-SEM enabled us to determine the spatial relationship between individual signals, by providing a direct measure of interparticle separation as opposed to indirect co-localization estimates or mean intensity values from fluorescence microscopy. Using this approach, we measured a significant decrease in the minimum mark separation distance from 254 ± 100 nm on flat substrates compared to 217 ± 74 nm on microgrooves (Figure 3f).

H3K9me3 is known to be enriched in heterochromatin, typically anchored by the nuclear lamina at the nuclear periphery.^[25]^ Thus, we sought to investigate whether the observed increase in H3K9me3 density correlated with its association at the nuclear lamina. For this analysis, we segmented the nuclei into a peripheral region adjacent to the nuclear envelope and a central region comprising the rest of the nucleus (Figure 3g). Peripheral regions of 150 nm in thickness were selected based on the knowledge that the lamina-anchored heterochromatin is located 30–100 nm below the nuclear membrane,^[26]^ which itself is 50 nm thick. The ability to volumetrically segment and subdivide regions of interest with such high resolution is currently not possible with optical microscopy techniques. This analysis revealed a significant increase in peripheral H3K9me3 immunolabels per cubic micrometer for nuclei on microgrooved substrates (16 ± 7) compared to flat substrates (7 ± 4), but no corresponding increase was observed in the central regions (24 ± 12 microgrooves vs 14 ± 9 flat) (Figure 3h). These results indicate that the microgroove-associated increase in H3K9me3 density stems from a preferential increase in H3K9 methylation at the nuclear periphery (Figure 3i). Consistent with the invariance of nuclear volume and the increase of histone marks in the periphery, we measured an increase in H3K9me3 density at the nuclear periphery exclusively for cells cultured on microgrooves, compared to the flat substrate (Figure 3j). This indicated a closer packing of peripheral H3K9me3 signals rather than an increase in peripheral volume through nuclear remodeling. The internal consistency of our results was validated by the observed increase in H3K9me3 density exclusively in the nuclear periphery when measured by counting immunogold labels per unit volume. Taken together, these data are consistent with the hypothesis that H3K9me3 may contribute to heterochromatin accumulation and gene silencing at the nuclear periphery, which occur during cell differentiation.^[27]^

We next investigated the effect of myoblast differentiation on nuclear shape and NPC distribution. Nuclear shape is known to directly influence gene expression and differentiation state; specifically, inward invagination of the inner and outer nuclear membranes is thought to provide access channels to the interior of the nucleus.^[28]^ Moreover, the inwardly invaginating double membrane has been speculated to contain a high density of NPCs, which facilitate the import and export of protein and messenger RNA (mRNA) complexes from gene active regions.^[29]^ Thus, we sought to investigate whether the requirements for changes in gene regulation during myogenesis were associated with nuclear shape changes and subsequent redistribution of NPCs. Murine myoblasts (C2C12 line) grown on the surface of gelatin methacryloyl (GelMA) hydrogel substrates were differentiated into myotubes using a 7 day culture in low-serum myogenic media supplemented with insulin-like growth factor-1.^[30]^ Undifferentiated myoblasts (day 0) and differentiated myotubes (day 7) were fixed and labeled with NPC-specific primary antibody and immunogold secondary antibody. Successful immunolabeling was confirmed using confocal fluorescence microscopy, which also revealed a relatively higher level of NPCs in myotubes compared to myoblasts (Figure 4a; Figure S2, Supporting Information).

We applied the immunogold FIB-SEM workflow to this system, confirming similar differences in NPC number, with a lower number of immunolabeled NPCs observed in the nuclei of myoblasts (53 ± 6) compared to myotubes (60 ± 12) (Figure S3, Supporting Information). We could also visualize well-defined ultrastructural features including the nuclear membrane, chromatin domains, and clear invaginations of both the outer and inner nuclear membranes (Figure 4b). These nanoscale invaginations, commonly referred to as type II nucleoplasmic reticulum (type II NR),^[28]^ are thought to improve access to the nuclear interior, which is typically rich in active genes.^[31]^ Interestingly, volumetric reconstructions revealed that the myotubes frequently exhibited deep inward invaginations in the form of type II NR (13 ± 4 per nucleus section), to a significantly greater level than was observed for myoblasts (3 ± 2) (Figure 4c,d). We next investigated whether NPCs were relocated to sites of nuclear invaginations by quantifying the percentage of NPCs localized at sites of type II NR. Interestingly, we also measured a significantly greater percentage of NPCs associated with type II NR in myotubes (43% ± 25%) compared to myoblasts (9% ± 7%) (Figure 4e). Our results are consistent with a few TEM studies that have alluded to the presence of NPCs at sites of type II NR.^[29,32]^ These data represent an unprecedented nanoscale 3D mapping of NPCs that reveals a role for NPC localization at type II NR in myogenesis.

Overall, our approach of combining FIB-SEM with immunogold labeling offers a new route to acquiring functional and structural information with volumetric nanoscale resolution (10 nm in plane, 90 nm out of plane). This strategy allowed us to map multiparticle population descriptors with nanoscale resolution (e.g., average nearest neighbor distance), offering a route to understanding the functionality of large molecular populations whose mutual relationship is of importance (e.g., clustering receptors, protein relocation, and proximity to ultrastructural features). In addition, our strategy mitigates counting issues that can arise from the low efficiency of immunogold labeling, by increasing the number of measured events and the overall completeness of sampling. In the future, however, this approach could be extended to other labeling techniques, such as the use of quantum dots, which could provide higher efficiency labeling.^[33]^ Moreover, it was possible to segment subcellular and suborganellular regions with unprecedented precision and evaluate the association of markers within these regions, enabling location–function correlation for key cellular regulators. Indeed, we used this technique to provide an advanced analysis of nuclear marker distribution in relation to nuclear ultrastructure. Specifically, we highlighted the preferential association of histone mark H3K9me3 with the nuclear lamina at the nuclear periphery of cells cultured on microgrooves and revealed that myoblast differentiation leads to significant reorganization in nuclear structure with NPCs localized to inward invaginations formed at the nuclear double membrane. These results demonstrate the versatility and unparalleled insight that immunogold FIB-SEM can provide when correlating cellular ultrastructure and biomolecular localization to provide insights into biological function, features that promise to make it a key analytical technique for dissecting the complex interplay between environmental cues, cell structure, and function in a broad range of applications.

## Supplementary Material

Supporting Information is available from the Wiley Online Library or from the author.

Supplementary Information

## Figures and Tables

**Figure 1:**
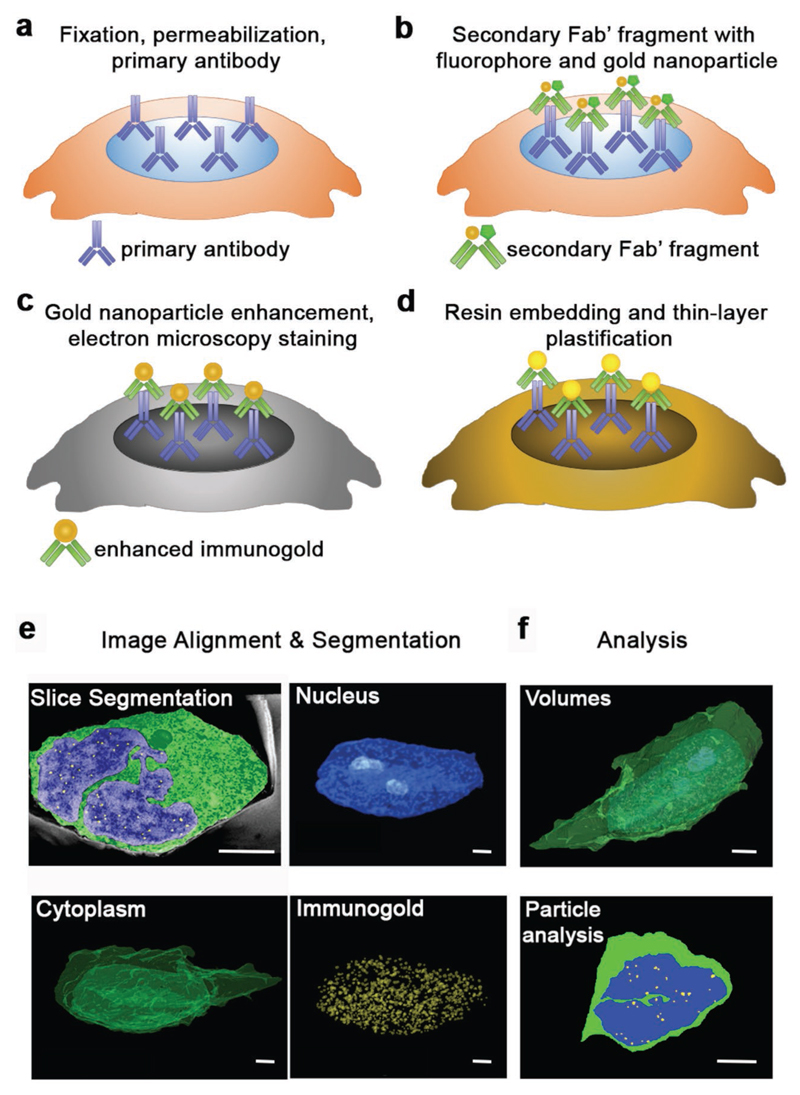
Schematic of the sample preparation workflow for immunogold FIB-SEM, combining processes for immunolabeling and electron microscopy. a) Cells are fixed, permeabilized, and incubated with a primary antibody of choice. b) The sample is next stained using an appropriate FluoroNano-Gold secondary antibody, bearing a 1.4 nm gold nanoparticle and an AlexaFluor dye. After this step, the samples can be imaged using fluorescence microscopy, if required. c) Next, the 1.4 nm gold nanoparticle on the secondary antibody is enhanced to a desired size (in this case 20–30 nm), after which samples are postfixed and taken through electron microscopy staining and dehydration. d) Finally, samples are infiltrated with resin, washed, and then samples are polymerized, ready for FIB-SEM imaging. e) FIB-SEM serial cross sections are aligned and regions of interest, such as the cytoplasm, nucleus, or other features such as immunogold labels of the antigen, are segmented from each slice. f) Analysis of particle number, distances, volumes of the segmented areas can then be conducted independently or in relation to each other. Scale bars = 2 μm.

**Figure 2:**
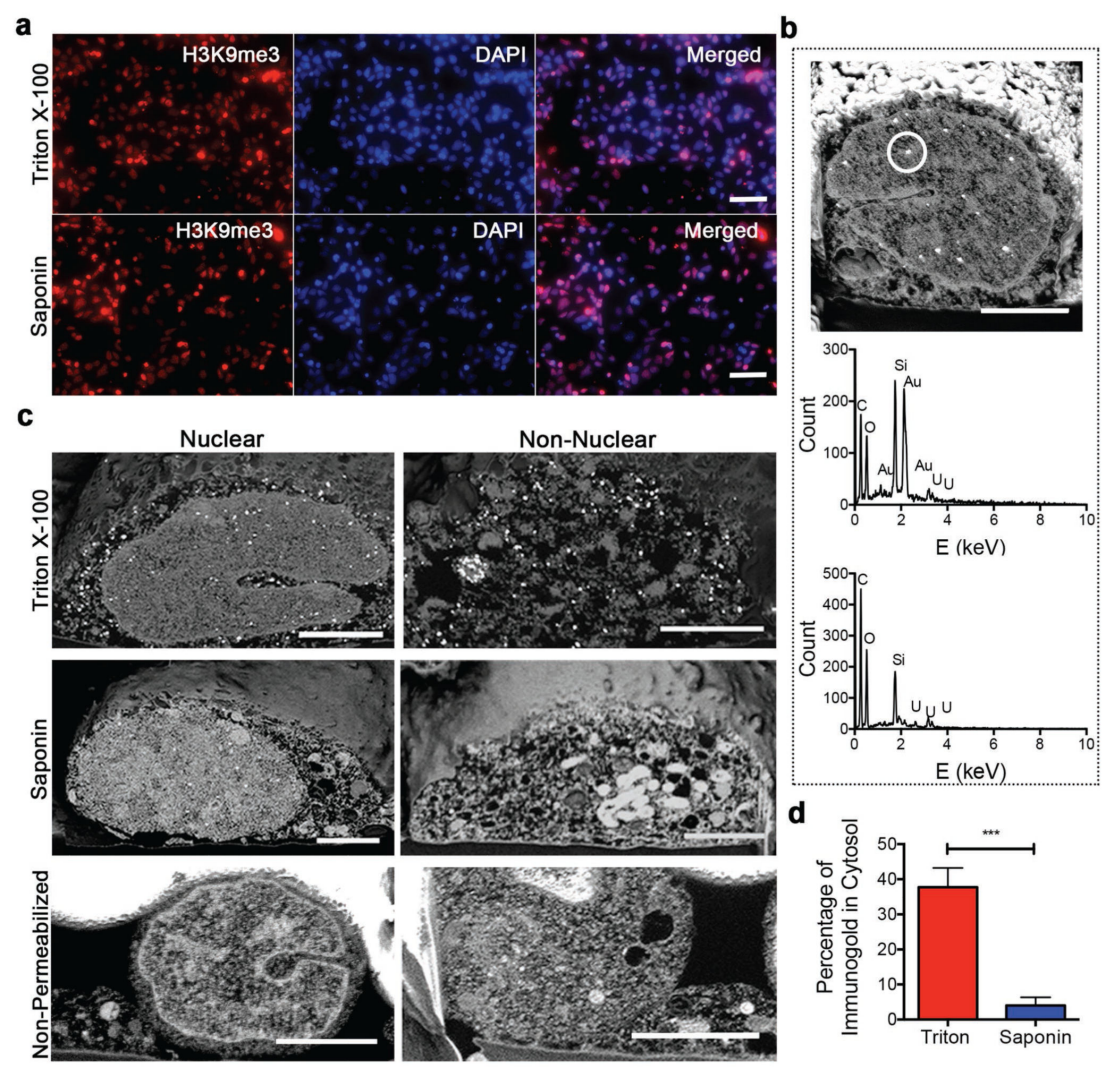
Optimization of immunogold FIB-SEM. a) Widefield fluorescence images of stem cells stained with DAPI, H3K9me3 primary antibody, and FluoroNanoGold secondary antibody after permeabilization with either Triton X-100 or Saponin. Scale bars = 20 μm. b) FIB-SEM cross section of a neural stem cell nucleus immunolabeled and prepared according to the workflow (top), with corresponding EDX spectra of the circled region indicating the presence of gold (middle). EDX spectra of a negative control with no H3K9me3 primary antibody added showed no gold present (bottom). Scale bars = 2 μm. c) Nuclear and non-nuclear FIB-SEM cross sections of neural stem cells either permeabilized with Triton X-100 or Saponin or not permeabilized at all. Scale bars = 2 μm. d) Quantification of immunogold particles for nuclear antigen H3K9me3 in the cytosol of samples permeabilized with Triton X-100 and Saponin as a percentage of the total visible labels. Plot shows mean ± standard deviation (S.D.), *n* = 3 (cells), *** *p* < 0.001 (two-tailed Mann–Whitney test).

**Figure 3:**
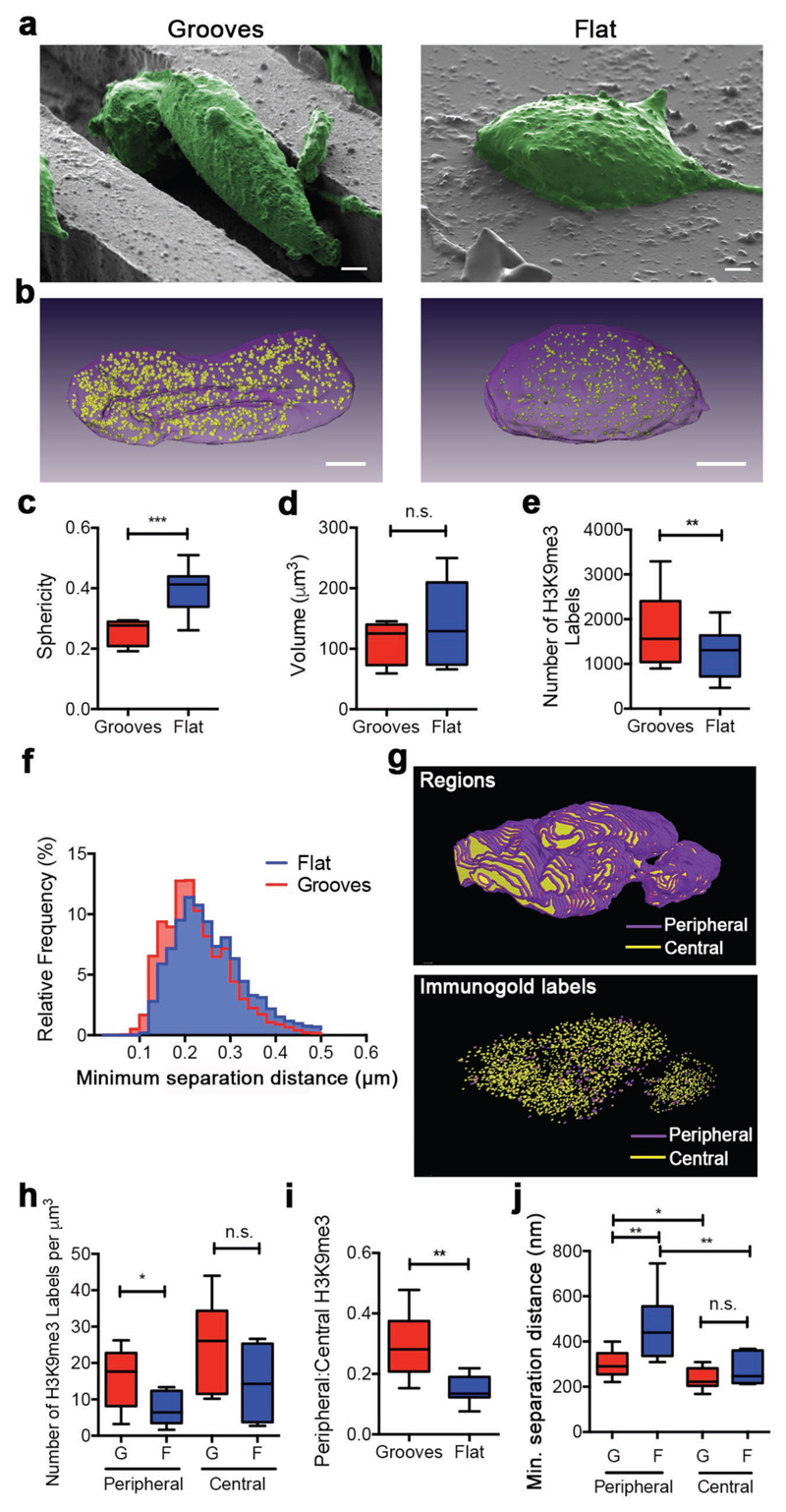
Immunogold FIB-SEM analysis of the epigenetic mark H3K9me3 during micro-groove-induced neuronal differentiation. a) False-colored SEM images of cells (green) on microgrooves and flat substrates prior to milling. Scale bars = 2 μm. b) 3D reconstruction of the nucleus (purple) and H3K9me3 immunolabels (yellow) within a cell cultured on micro-grooved topography or flat PDMS substrate. Scale bars = 2 μm. c) Quantification of 3D sphericity. d) Quantification of the volume of the nuclei of cells cultured on microgrooves or flat substrates. Data presented as min-to-max plots, *n* = 9 (cells), *** *p* < 0.001, two-tailed Mann–Whitney nonparametric test. n.s. = Not significant. e) Quantification of H3K9me3 immunolabels within nuclei of cells cultured on microgrooved and flat PDMS substrates. Data presented as min-to-max plots, *n* = 9 (cells), ** *p* < 0.01, two-tailed Wilcoxon paired nonparametric test. f) Relative frequency histogram of minimum separation distance between H3K9me3 immunogold pairs in nuclei of cells on microgrooves and flat surfaces. g) Representative 3D reconstruction of the nucleus of a cell cultured on microgrooves with segmented peripheral regions (purple, up to 150 nm from the nuclear surface) and central regions (yellow). H3K9me3 immunogold labels within the same nucleus were segmented based on their positioning being either central or peripheral. h) Quantification of peripheral and centrally located H3K9me3 immunogold labels for nuclei of cells on microgrooves and flat substrates per cubic micrometer. Data presented as min-to-max plots, *n* = 9 (cells), * *p* < 0.05, two-tailed Mann–Whitney nonparametric test. n.s. = Not significant. G = Microgrooves, F = Flat. i) Ratio of peripheral-to-central H3K9me3 immunogold labels in nuclei of cells on microgrooves and flat substrates. Data presented as min-to-max plots, *n* = 9 (cells), ** *p* < 0.01, two-tailed Mann–Whitney nonparametric test. j) Quantification of minimum separation distance of immunogold labels as a function of location (periphery or center) and substrate (microgrooves or flat). Data presented as min-to-max plots, *n* = 9 (cells), * *p* < 0.05, ** *p* < 0.01, two-tailed Mann–Whitney non-parametric test. n.s. = Not significant. G = Microgrooves, F = Flat.

**Figure 4:**
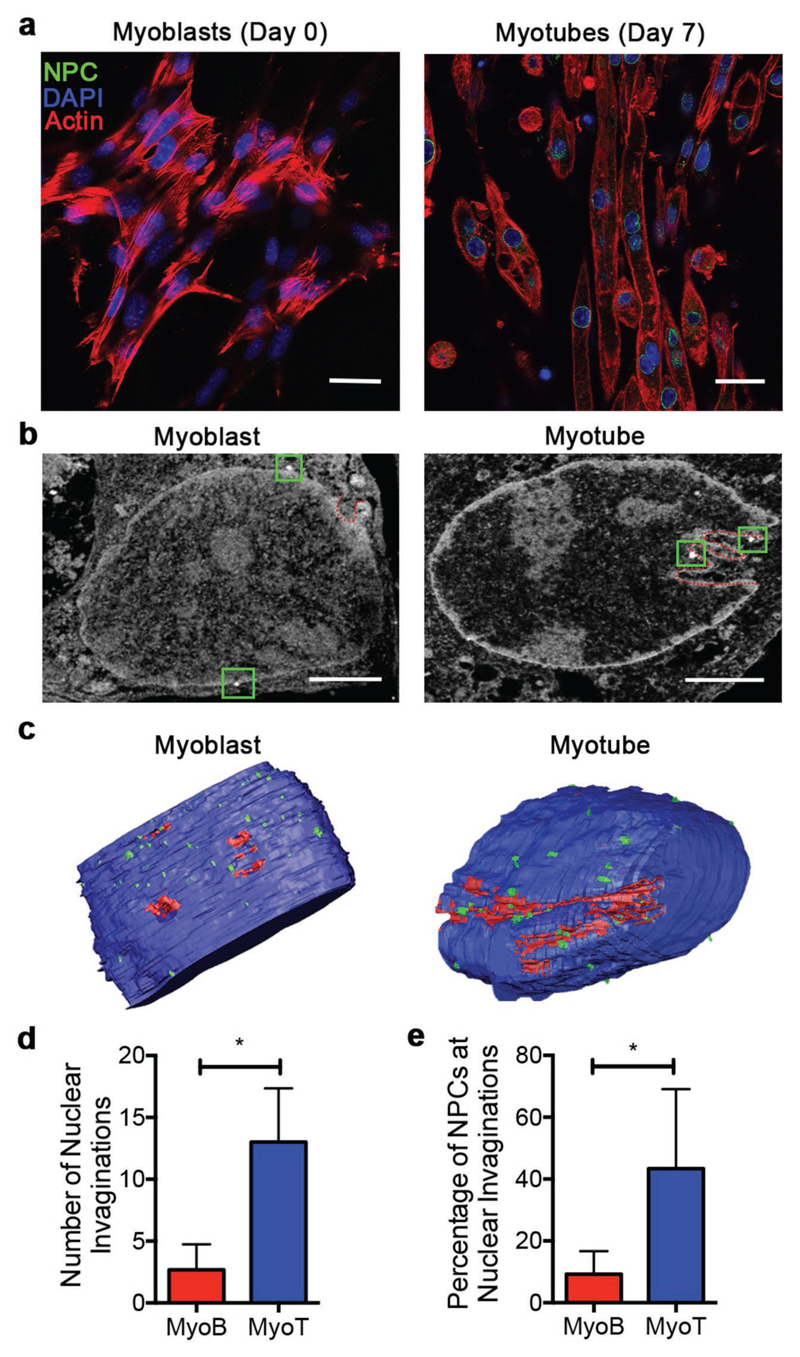
Immunogold FIB-SEM analysis of NPC localization before and after myogenesis. a) Confocal fluorescence microscopy of myoblasts and myotubes labeled with NPC antibody mab414 (NPC, green), DAPI (DNA, blue), and phalloidin (actin, red). Scale bars = 20 μm. b) Representative immunogold FIB-SEM cross sections of a myoblast and a myotube, showing immunolabeled NPCs (green squares) and nuclear folds (red dotted lines). Scale bars = 2 μm. c) 3D reconstructed sections of nuclei (blue) with segmented folds (red) and immunolabeled NPC (green) for myoblasts and myotubes. d) Quantification of the number of nuclear invaginations in myoblasts and myotubes. e) Quantification of the number of NPCs present at nuclear invaginations in myoblasts and myotubes. Plots show mean ± S.D., *n* = 3–4 (cells), at least 50 FIB-SEM cross sections per cell, * *p* < 0.05, two-tailed Mann–Whitney test. MyoB = Myoblasts, MyoT = Myotubes.

## References

[1] Lin ZC, McGuire AF, Burridge PW, Matsa E, Lou H-Y, Wu JC, Cui B (2017). Microsyst Nanoeng.

[2] Dalby MJ, Gadegaard N, Wilkinson CDW (2008). J Biomed Mater Res, Part A.

[3] Engler AJ, Sen S, Sweeney HL, Discher DE (2006). Cell.

[4] Yamamoto K, Sokabe T, Watabe T, Miyazono K, Yamashita JK, Obi S, Ohura N, Matsushita A, Kamiya A, Ando J (2005). Am J Physiol: Heart Circ Physiol.

[5] McKee C, Hong Y, Yao D, Chaudhry GR (2017). Tissue Eng, Part A.

[6] Guan J, Wang F, Li Z, Chen J, Guo X, Liao J, Moldovan NI (2011). Biomaterials.

[7] Schermelleh L, Heintzmann R, Leonhardt H (2010). J Cell Biol.

[8] Tam J, Merino D (2015). J Neurochem.

[9] Wegel E, Göhler A, Lagerholm BC, Wainman A, Uphoff S, Kaufmann R, Dobbie IM (2016). Sci Rep.

[10] Maglione M, Sigrist SJ (2013). Nat Neurosci.

[11] Kizilyaprak C, Daraspe J, Humbel BM (2014). J Microsc.

[12] Santoro F, Zhao W, Joubert L-M, Duan L, Schnitker J, van de Burgt Y, Lou H-Y, Liu B, Salleo A, Cui L, Cui Y (2017). ACS Nano.

[13] Persson H, Købler C, Mølhave K, Samuelson L, Tegenfeldt JO, Oredsson S, Prinz CN (2013). Small.

[14] Chiappini C, Martinez JO, De Rosa E, Almeida CS, Tasciotti E, Stevens MM (2015). ACS Nano.

[15] Gopal S, Chiappini C, Penders J, Leonardo V, Seong H, Rothery S, Korchev Y, Shevchuk A, Stevens MM (2019). Adv Mater.

[16] Collinson LM, Carroll EC, Hoogenboom JP (2017). Curr Opin Biomed Eng.

[17] Porter AE, Muller K, Skepper J, Midgley P, Welland M (2006). Acta Biomater.

[18] Fiserova J, Spink M, Richards SA, Saunter C, Goldberg MW (2014). J Cell Sci.

[19] Schnell U, Dijk F, Sjollema KA, Giepmans BNG (2012). Nat Methods.

[20] Hannah MJ, Weiss U, Huttner WB (1998). Methods.

[21] Miles BT, Greenwood AB, Benito-Alifonso D, Tanner H, Galan MC, Verkade P, Gersen H (2017). Sci Rep.

[22] Downing TL, Soto J, Morez C, Houssin T, Fritz A, Yuan F, Chu J, Patel S, Schaffer DV, Li S (2013). Nat Mater.

[23] Morez C, Noseda M, Paiva MA, Belian E, Schneider MD, Stevens MM (2015). Biomaterials.

[24] Dillon N (2008). Dev Cell.

[25] Becker JS, Nicetto D, Zaret KS (2016). Trends Genet.

[26] Carroll M (1989). Organelles.

[27] Mattout A, Cabianca DS, Gasser SM (2015). Genome Biol.

[28] Malhas A, Goulbourne C, Vaux DJ (2011). Trends Cell Biol.

[29] Goulbourne CN, Malhas AN, Vaux DJ (2011). J Cell Sci.

[30] Armstrong JPK, Puetzer JL, Serio A, Guex AG, Kapnisi M, Breant A, Zong Y, Assal V, Skaalure SC, King O, Murty T (2018). Adv Mater.

[31] Schoen I, Aires L, Ries J, Vogel V (2017). Nucleus.

[32] Wittmann M, Queisser G, Eder A, Wiegert JS, Bengtson CP, Hellwig A, Wittum G, Bading H (2009). J Neurosci.

[33] Kuipers J, de Boer P, Giepmans BNG (2015). Exp Cell Res.

