# Supplementary material for: Immunogold FIB-SEM: Combining Volumetric Ultrastructure Visualization with 3D Biomolecular Analysis to Dissect Cell–Environment Interactions
Source: Adv Mater. Author manuscript; Available in PMC 2019 Oct 4. (PMC6778054; doi:10.1002/adma.201900488)
Supplement: Supplementary Information [file EMS83593-supplement-Supplementary_Information.docx]

**SUPPORTING INFORMATION**

Immunogold FIB-SEM: combining volumetric ultrastructure visualization with 3D biomolecular analysis to dissect cell-environment interactions

**Sahana Gopal**^1,2^, Ciro Chiappini^1,3,†^, James P. K. Armstrong^1^, Qu Chen^1^, Andrea Serio^1,†^, Chia-Chen Hsu^1,‡^, Christoph Meinert^4^, Travis J. Klein^4,5^, Dietmar W. Hutmacher^4,5^, Stephen Rothery^6^, Molly M. Stevens^1^

^1^Department of Materials, Department of Bioengineering and Institute of Biomedical Engineering, Imperial College London, London SW7 2AZ

^2^Department of Medicine, Imperial College London, London, W12 0NN

^3^Centre for Craniofacial and Regenerative Biology, King’s College London, London, SE1 9RT

^4^Institute of Health and Biomedical Innovation and Australian Research Council Training Centre in Additive Biomanufacturing, Queensland University of Technology, Brisbane, Queensland 4059, Australia

^5^Australian Research Council Industrial Transformation Training Centre, Queensland University of Technology, Brisbane, Queensland 4059, Australia

^6^Facility for Light Microscopy, Imperial College London, London, SW7 2BB

**Present Address**

†Centre for Craniofacial and Regenerative Medicine, King’s College London, SE1 9RT

‡Institute of Biomedical Engineering, Department of Engineering Science, University of Oxford, OX3 7DQ

MATERIALS AND METHODS

**Cell Culture and Seeding**

Neural stem cells (NSCs) were derived from human induced pluripotent stem cells (hiPSCs) using a previously validated protocol^[1]^, which itself was modified from a protocol of Bilican *et al.*^[2]^. The hiPSCs were differentiated into neuroectoderm by dual SMAD signalling inhibition^[3]^, using Neural Basal Medium consisting of Advanced DMEM/F-12 medium (Thermo Fisher Scientific), 1% (v/v) N-2 supplement (Invitrogen), 0.2% (v/v) B27 Supplement (Invitrogen), 1% (v/v) penicillin/streptomycin (Invitrogen) and 1% (v/v) GlutaMAX (Invitrogen). The Neural Basal Medium was supplemented with SB431542 (10 μM; Calbiochem) and InSolution™ AMPK Inhibitor, Compound C (2 μM; Calbiochem) for 7−10 days. After enzymatic dissociation, hiPSC-derived NSCs were passaged and seeded on plates coated with laminin (Sigma-Aldrich). After 3−5 days culture in Neural Basal Medium, the NSCs proliferated and formed neural rosette structures and were then maintained in Neural Basal Medium supplemented with 20 ng/mL fibroblast growth factor 2 (PeproTech). NSCs were usually subcultured every 5−7 days on laminin-coated plates for the first few passages and on culture plates coated with Matrigel (Corning) for later passages. NSCs were detached with Accutase (Stemcell Technologies) when the cells were more than 80% confluent. The dissociated NSCs were suspended in PBS, pelleted at 300 x g for 5 minutes and resuspended into single cells in Neural Basal Medium supplemented with 10 ng ml^-1^ brain-derived neurotrophic factor (R&D Systems), 10 ng ml^-1^ glial cell line derived neurotrophic factor (R&D Systems), and forskolin (10 μM; Sigma-Aldrich). NSCs were then plated on substrates placed within a 48-well plate at a density of 6.6 x 10^4^ cells cm^-2^, cultured for 2 days and fixed for immunostaining.

All reagents for murine myoblast culture were purchased from Thermo Fisher Scientific unless otherwise stated. Murine myoblasts (C2C12 line, ATCC) were expanded in myoblast culture medium: high glucose Dulbecco’s Modified Eagle’s Medium (DMEM) with 1% (v/v) penicillin/streptomycin and 20% (v/v) fetal bovine serum. Myoblasts were detached with Trypsin/EDTA solution when the cells were 60-70% confluent, pelleted at 400 x g for 4 minutes and resuspended into a single cell suspension in myoblast culture medium. Myoblasts were seeded at a density of 2.0 x 10^4^ cells cm^-2^ on 500 µL 4% gelMA hydrogels pre-cast in a 24-well plate. The cell-seeded constructs were cultured for three days in myoblast culture medium before initiating myogenesis using myogenic differentiation medium: high glucose DMEM with 1% (v/v) penicillin/streptomycin, 1% N2 supplement, 1% non-essential amino acids and 20 ng L^-1^ of freshly-supplemented recombinant human IGF-1 (PeproTech). The medium was changed every day for a differentiation period of seven days.

**Immunolabeling**

HiPSC-derived NSCs were fixed with 4% (v/v) PFA (Sigma-Aldrich) in PBS for 15 minutes, permeabilized with 0.1% (v/v) Triton X-100 or 0.1% (w/v) Saponin (Sigma-Aldrich) for 5 minutes at room temperature with either 0.1% (w/v) saponin (Sigma-Aldrich) in PBS or 0.1% (v/v) Triton X-100 (Sigma-Aldrich) in PBS. 0.1% (w/v) saponin was added for all subsequent blocking and antibody incubations in the saponin-permeabilized group. The samples were blocked for 30 minutes using 3% (v/v) goat serum (Thermo Fisher Scientific), 0.1% (w/v) saponin was added in PBS, then incubated for 1 hour with rabbit anti-H3K9me3 primary antibody (Abcam) diluted 1:1000 in 1.5% (v/v) goat serum in PBS. For pre-embedding immunogold labelling, samples were washed 3 times for 10 minutes using 5% (w/v) skimmed milk (Sigma-Aldrich) and 1% (v/v) goat serum in PBS and then incubated in the dark at room temperature for 90 minutes with AlexaFluor® 488 FluoroNanoGold™ anti-rabbit Fab’ fragment (Nanoprobes) diluted 1:100 in 1% (w/v) skimmed milk (and additional 0.1% saponin if saponin-treated sample) in PBS. Samples were washed four times for 10 minutes using PBS, and then mounted in Vectashield (Vector Labs) for fluorescence imaging, prior to immunogold enhancement.

Myoblasts/myotubes were fixed with 4% (v/v) PFA for 10 minutes, permeabilized for 5 minutes at room temperature with 0.1% (w/v) saponin in PBS. 0.1% (w/v) saponin was added for all subsequent blocking steps, antibody incubations and wash step. The samples were blocked overnight using 3% (v/v) bovine serum albumin (BSA, Sigma-Aldrich) in PBS, then incubated for 1 hour with nuclear pore complex rabbit anti-mab414 primary antibody (Abcam) diluted 1:10000 in 3% (v/v) BSA in PBS. For pre-embedding immunogold labeling, samples were washed and treated with FluoroNanoGold™ by the same procedure as described above. The samples were counterstained for 10 minutes using 4′,6-diamidino-2-phenylindole (DAPI, Thermo Fisher Scientific) and for 20 minutes using Alexa Fluor 555 Phalloidin (Thermo Fisher Scientific), then washed once with PBS prior to fluorescence imaging.

**Gold Enhancement and Electron Microscopy Preparation**

Gold Enhance EM Plus (Nanoprobes) was used for enhancement of the FluoroNanoGold™ nanoparticles, with all reagents were prepared according to the manufacturer’s instructions. After imaging, the samples were washed four times for 10 minutes using PBS, fixed for 10 minutes with 2.5% (v/v) glutaraldehyde (GA, EM Sciences) in PBS, and rinsed extensively in deionized water to remove any remaining salts. Gold enhance solution was applied on the samples for 5 minutes and then washed in double distilled water twice for 5 minutes. Samples were incubated in 0.1 M sodium cacodylate buffer (pH 7.4, EM Sciences) twice for 5 minutes and then treated with 1% (v/v) OsO_4_ in 0.1 M sodium cacodylate buffer for 1 hour at room temperature. Samples were washed in deionized water twice for 5 minutes and then incubated with 1% (w/v) tannic acid in deionized water for 1 hour. Samples were washed in deionized water twice for 5 minutes and incubated with 1% (w/v) filtered uranyl acetate (UA, EM Sciences) solution in deionized water for at least 150 minutes in the dark. Samples were dehydrated with ethanol through a series of steps (20, 30, 50, 70, 80, 90%) with each concentration of ethanol applied twice for 5 minutes each. Samples were incubated with 100% ethanol four times for 5 minutes. Epon resin embedding kit (Sigma Aldrich) was used to infiltrate the samples. Samples were incubated with excess increasing concentrations of Epon: ethanol (1:3, 1:2, 1:1) over a period of 3 hours each followed by 2:1 Epon:ethanol overnight. Samples were infiltrated with pure Epon twice for 3 hours each, and then excess resin was rinsed off with ethanol until only a thin layer of resin remained on the samples. Infiltrated samples were polymerized at 60 °C for 24-48 hours.

**Fabrication of PDMS Substrates**

Soft lithography molds made of parallel microgrooves (10 μm wide and 10 μm deep) were prepared by spinning SU-8 2002 at 1000 RPM for 40 s on a silicon wafer. The resist was pre-baked at 95 °C for 120 s on hotplate and exposed using a 10 μm stripe, 10 μm pitch photomask (K.Suss MA6 mask aligner). The substrate was post-exposure baked for 3 min at 95 °C on a hotplate and developed for 300 s in SU-8 developer. For the production of the PDMS replicas, silicone elastomer and curing agent (Sylgard 182 Kit, Dow Corning) were mixed at a ratio of 10:1 (v/v) and placed in a vacuum chamber for 30 minutes. The soft lithography molds were spin-coated with PDMS at 500 RPM for 40 seconds and cured at 130 °C for 15 minutes. The PDMS substrates were de-molded and cut to the appropriate size with a hole puncher. PDMS membranes were cut at the border between the microgrooves and flat substrate to ensure that each of the substrates consisted of both microgrooved and flat areas for cell seeding. The substrates were sonicated in 70% (v/v) ethanol for 20 minutes (VWR, 150T Sonicator), sonicator), dried and then treated with oxygen plasma for 1 minute (PlasmaPrep II, Gala Instrumente) to increase hydrophilicity and protein binding. Membranes were washed in 70% (v/v) ethanol, transferred to a 24 well culture plate (Corning), UV-sterilized for 1 hour and then coated with 2% (w/v) gelatin and 1% (w/v) laminin (Sigma Aldrich) at 37 °C for at least 30 minutes prior to cell seeding.

**Preparation of Gelatin Methacryloyl (GelMA) Hydrogels**

Gelatin methacryloyl (GelMA) with 88 ± 1 % amine functionalization was synthesized and characterized following previously published protocols^[4]^. A 4% (w/v) pre-gel was prepared by diluting lyophilized gelMA in a solution of sterile-filtered 5 mg mL^-1^ Irgacure 2959 (Sigma) in PBS at 60 °C. 500 µL of pre-gel solution was added to each well of a 24-well plate and then crosslinked using a 2-minute irradiation of UV (365 nm, 6 mW cm^-2^). Prior to cell seeding, the hydrogels were washed three times in PBS to remove any excess Irgacure 2959.

**FIB-SEM Operation**

Samples were sputtered with 10 nm chromium (Quorum Q150T S) prior to FIB-SEM imaging. Samples were imaged using a FIB-SEM (Cross Beam Working Station, Auriga, Zeiss). Regions of interest were located using a secondary electron secondary ion beam (SESI) at an accelerating voltage of 5 kV and a standard aperture of 30 μm. The stage was tilted to 54°, the acceleration voltage dropped to 1.6 kV and the sample milled with a current of 1 nA at a working distance of 5 mm. Sections were milled and imaged at a desired interval using an in-lens EBSD detector.

**Energy Dispersive X-ray (EDX) Spectroscopy**

EDX spectra were obtained using an AZtec Energy system and INCA software (Oxford Instruments) equipped within the FIB-SEM instrument. Pointwise spectra were collected by dwelling the electron beam at the desired spot. Spots with high electron scattering putatively associated with gold nanoparticles were analyzed, alongside control spots. The incident electron beam energy was set at 20 keV and the emitted X-ray collected over the 0-10 keV energy range, integrating for 60 seconds at every point. INCA software was used to analyze the relative atomic and weight composition of the selected elements within the analysis area.

**Image Analysis**

FIB-SEM images were aligned in Amira (FEI) using a combination of manual and automatic alignment. Regions of interest such as the nucleus or immunogold labels were segmented manually. Segmented label files were imported into ImageJ or Volocity (Perkin Elmer) for further analysis.

**Statistical Analysis**

Statistical analysis was performed using GraphPad Prism 6.0. Since the data were not normally distributed the non-parametric unpaired Mann-Whitney test was used. For quantification of immunogold labels, the paired Wilcoxon matched pairs signed rank test was used due to variations in immunogold labeling and enhancement between experiments. Significant differences were reported for p-values <0.05.

**
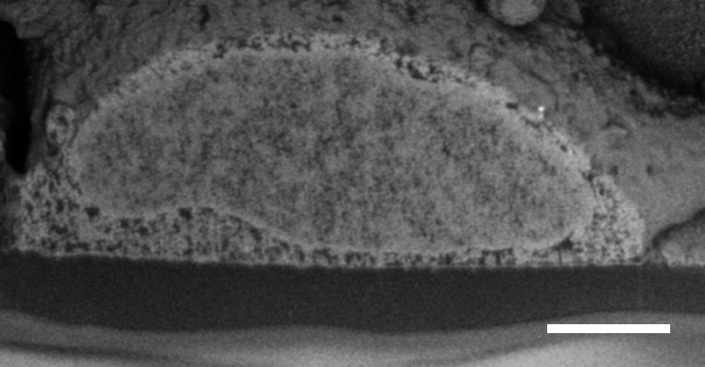
Supplementary Figures**

**Supplementary Figure 1** FIB-SEM cross-section of a neural stem cell prepared according to the workflow without primary antibody H3K9me3 immunolabeling (negative control). Scale bar = 2 µm.


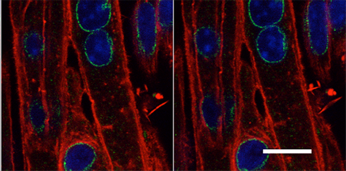

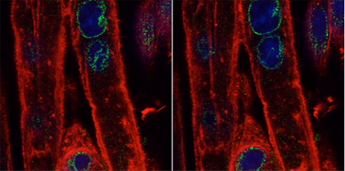


**Supplementary Figure 2** Serial confocal fluorescence microscopy images of myotubes labeled with NPC antibody mab414 (NPC, green), DAPI (DNA, blue) and phalloidin (actin, red) indicating multiple nuclei with nuclear folds. Scale bars = 20 µm.


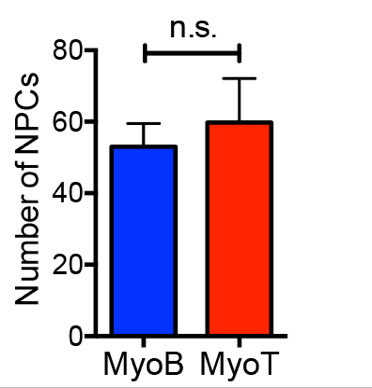


**Supplementary Figure 3** Quantification of the number of immunogold labelled NPCs in the nuclei of myoblasts (MyoB) and myotubes (MyoT). Plot shows mean ± S.D., n = 3-4 (nuclei), at least 50 nuclear FIB-SEM cross-sections per cell, p>0.05, n.s., two-tailed Mann-Whitney non-parametric test.

References

[1] C.-C. Hsu, A. Serio, N. Amdursky, C. Besnard, M. M. Stevens, *ACS Appl. Mater. Interfaces* **2018**, *10*, 5305.

[2] B. Bilican, A. Serio, S. J. Barmada, A. L. Nishimura, G. J. Sullivan, M. Carrasco, H. P. Phatnani, C. A. Puddifoot, D. Story, J. Fletcher, I.-H. Park, B. A. Friedman, G. Q. Daley, D. J. A. Wyllie, G. E. Hardingham, I. Wilmut, S. Finkbeiner, T. Maniatis, C. E. Shaw, S. Chandran, *Proc. Natl. Acad. Sci. U.S.A.* **2012**, *109*, 5803.

[3] S. M. Chambers, C. A. Fasano, E. P. Papapetrou, M. Tomishima, M. Sadelain, L. Studer, *Nat. Biotechnol.* **2009**, *27*, 275.

[4] D. Loessner, C. Meinert, E. Kaemmerer, L. C. Martine, K. Yue, P. A. Levett, T. J. Klein, F. P. W. Melchels, A. Khademhosseini, D. W. Hutmacher, *Nat Protoc* **2016**, *11*, 727.
